# *Trypanosoma lewisi* infections in *Rattus rattus* from rural ecosystems in Gabon

**DOI:** 10.1016/j.ijppaw.2026.101257

**Published:** 2026-06-20

**Authors:** Clark Mbou-Boutambe, Larson Boundenga, Carine Brouat, Philippe Gauthier, Odile Fossati-Gaschignard, Fanny Degrugillier, Cyr Moussadji, Thierry Audrey Tsoumbou, Barthélemy Ngoubangoye, Virginie Rougeron, Laurent Granjon, Franck Prugnolle

**Affiliations:** a Ecole Doctorale Régionale d'Afrique Centrale en Infectiologie Tropicale (EDR), BP 876, Franceville, Gabon; b Unité de Recherche en Écologie de la Santé (URES), Centre Interdisciplinaire de Recherches Médicales de Franceville (CIRMF), BP 769, Franceville, Gabon; c Département d'Anthropologie, Université de Durham, South Road, Durham, DH1 3LE, UK; d CBGP, IRD, CIRAD, INRAE, Institut Agro, Université de Montpellier, Montpellier, France; e Institut de Recherche pour le Développement (IRD), Maladies Infectieuses et Vecteurs, Écologie, Génétique, Évolution et Contrôle (MIVEGEC) (Université de Montpellier-IRD 224-CNRS 5290), Montpellier, 34394, France; f Centre de Primatologie, Centre Interdisciplinaire de Recherches Médicales de Franceville (CIRMF), BP 769, Franceville, Gabon; g International Research Laboratory-REHABS, CNRS-Université Lyon 1-Nelson Mandela University, Nelson Mandela University George Campus, George, 6531, South Africa

**Keywords:** Rodents, *Trypanosoma lewisi*, *Herpetosoma,* Africa and zoonosis

## Abstract

Rodents are important reservoirs for numerous zoonotic parasites. Among them, *Trypanosoma lewisi* (subgenus *Herpetosoma*) has been introduced into many regions worldwide through its primary reservoir, *Rattus*, which has spread globally along human commercial routes. Several other *Herpetosoma* species have been reported to infect native rodents in Africa, although their zoonotic potential remains unclear. In rural areas of Gabon, *R. rattus* is widely distributed within villages, being largely dominant in rodent communities. Based on sampling conducted across 11 localities, we screened 528 rodents.

Using qPCR targeting the 18S rDNA gene. The overall prevalence for this subgenus was 56.6% (n = 299). *Trypanosoma* spp. were detected at all sampling sites, with prevalence in *R. rattus* ranging from 21.6% to 85.7% in domestic localities. Infections were also observed in *Praomys jacksoni* (100%), *Lophuromys roseveari* (72%), *Lemniscomys striatus* (25%), and *Hybomys univittatus* (71%). To formally identify the circulating species, a 2100 bp fragment of the 18S rDNA gene was successfully sequenced from a subset of 21 qPCR-positive samples, revealing that all these characterized isolates belonged to the subgenus *Herpetosoma* and clustered tightly within the *T. lewisi* subclade (genotype AF05b), recognized as *T. lewisi* sensu stricto. Our findings confirm the occurrence of *T. lewisi* AF05b within invasive *R. rattus* populations in rural Gabon. While *T. lewisi* DNA was also detected in a few native rodents, the low trypanosome diversity observed in this study is likely driven by the overwhelming dominance of *R. rattus* in our sampling. This study underscores the widespread presence of the *T. lewisi* clade in invasive rats in Gabon and emphasizes the importance of monitoring invasive rodent populations as potential amplifiers of zoonotic trypanosomes, though further targeted studies with balanced sampling are required to clarify the exact epidemiological role of native African rodents in these transmission cycles.

## Highlights

• Trypanosome infections were detected in all eight rural localities studied in Gabon, covering domestic, peri-domestic, and forest environments.
• First documentation of *Trypanosoma lewisi* in *Rattus* in Gabon.
• The black rat (*Rattus*) accounts for 89% of all infections, confirming its role as the primary reservoir and dispersal host for *T. lewisi* in Central Africa.

## Graphical abstract

**Figure:**
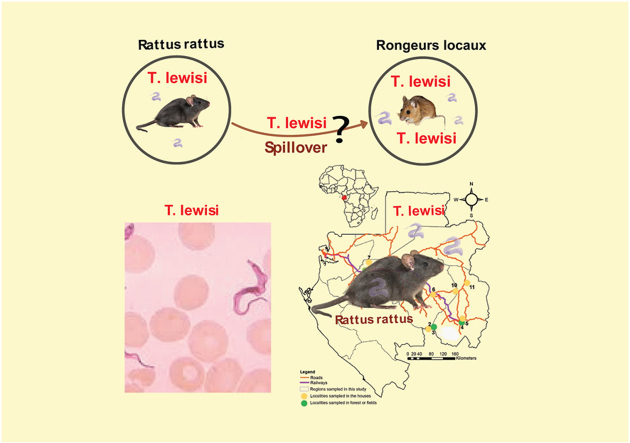

## 1 Introduction

Rodents constitute the most abundant and diverse group of mammals, and are known to be natural reservoirs of multiple pathogens (Han et al., 2016). Owing to their high population densities, wide environmental distribution, and the synanthropic behavior of several globally widespread species such as rats or mice, rodents play a key role in the transmission of zoonotic diseases to humans and domestic animals (Han et al., 2016; Dahmana et al., 2020). Human-driven global changes, including deforestation and agricultural expansion, can increase the risk of zoonotic disease emergence by bringing humans, livestock, and wild animals including rodents into closer contact (Meerburg et al., 2009; White and Razgour, 2020). Spillover events are particularly likely when conditions promote rodent population growth, thereby intensifying interactions among rodents, humans and the parasites they harbour (Meerburg et al., 2009; Morand et al., 2015). Additionally, the introduction of exotic rodent species that are often synanthropic and benefit from human constructions and activities for shelter and food may influence zoonotic risk, by spreading pathogens, displacing native rodent hosts, or serving as bridge hosts between wild native rodents and humans or domesticated animals (Morand et al., 2015; DiagnE et al., 2016; Roy et al., 2023).

Among the parasites carried by rodents that may pose a threat to humans and domestic animals are protozoans of the genus *Trypanosoma*. These trypanosomes, primarily transmitted by hematophagous vectors, infect a wide range of vertebrate hosts, including humans, wildlife, and domestic animals (Boundenga et al., 2022; Cassan et al., 2018; Cayla et al., 2019). Well-known species include *Trypanosoma cruzi* in the Americas, responsible for Chagas disease (Teixeira et al., 2018), as well as various African subspecies of *Trypanosoma brucei*: *T. brucei gambiense*, which causes the chronic form of human African trypanosomiasis (sleeping sickness), *T. brucei rhodesiense*, responsible for a more acute and often fatal form, and *T. brucei*, which causes African animal trypanosomiasis (nagana) in livestock, leading to significant economic losses (Truc et al., 2013; Büscher et *al*., 2017).

Host specificity varies widely among *Trypanosoma* spp., from extremely low in *T. cruzi* (which infect several mammalian species including humans) to species confined to a single mammal host (Hoare, 1967). Rodent trypanosomes from the subgenus *Herpetosoma* are typically considered as highly host-specific (Votýpka et *al*., 2022). The only exception is *T*. *lewisi*, whose cosmopolitan distribution in rodents has been linked to the global, human-mediated spread from Asia of its primary hosts, rats of the genus *Rattus,* and has now been reported in more than 100 rodent species worldwide (Votýpka et *al*., 2022). *Trypanosoma lewisi* was suspected to have played a role in the extinction of two endemic rat species on Christmas Island following the introduction of black rats (*Rattus*) (Wyatt et al., 2008). Initially considered as non-pathogenic to humans (Truc et al., 2013), this species was nevertheless reported in several cases of human infection in Africa (Howie et al., 2006) and Asia (Liu and Liu, 1990, Kaur et al., 2007; Verma et al., 2011; Shah et al., 2011; Doke and Kar, 2011).

Several surveys documented *Trypanosoma* infection in rodents of Sub-Saharan Africa. Some of these studies focused on urban environments, and hence showed the occurrence of *T. lewisi*, especially in villages and cities invaded by *R. rattus* (Dobigny et al., 2011; Tatard et al., 2017; Salzer et al., 2016; Mangombi et al., 2021; Cassan et al., 2018; Votýpka et al., 2022; Babyesiza et al., 2024). Nevertheless, studies conducted in rural or wild areas and using more resolutive molecular markers evidenced several other species of *Trypanosoma* in native rodents, including numerous *T. lewisi*-like, revealing an unexpected diversity (Votýpka et *al*., 2022).

In this study, we focused on rodent communities in rural areas of Gabon. Like in many other African countries, rural ecosystems of Gabon, and notably villages, are invaded by the exotic *R. rattus* to the detriment of native rodents (Mbou-Boutambe et al., 2024). Some native species may co-occur with *R. rattus*, but some others occur exclusively in more natural habitats. Our aim was to screen exotic and native rodents for *Trypanosoma*, in order to characterize the diversity of these parasites and the distribution of the zoonotic species *T. lewisi*.

## 2 Material and methods

### 2.1 Ethical statement

Rodent trapping was authorized by the scientific commission for the examination and application for research authorisation of Gabon (N°AR005/20/MESRSTT/CENAREST/CG/CST/CSAR). The capture, handling, and necropsy of animals were carried out in accordance with the guidelines of the American Society of Mammalogists (Sikes, 2016) and in strict compliance with the recommendations of the Ethics Committee for Animal Experimentation of Languedoc Roussillon No. 36 (reference number 2020-02 v3). The samples were transferred to France for further analysis in agreement with Gabon documentation and immigration service (N°/MIDSHP/FPN/DGDI/AR/H.O/FCV).

### 2.2 Study sites, rodent trapping and identification

**Fig. 1:**
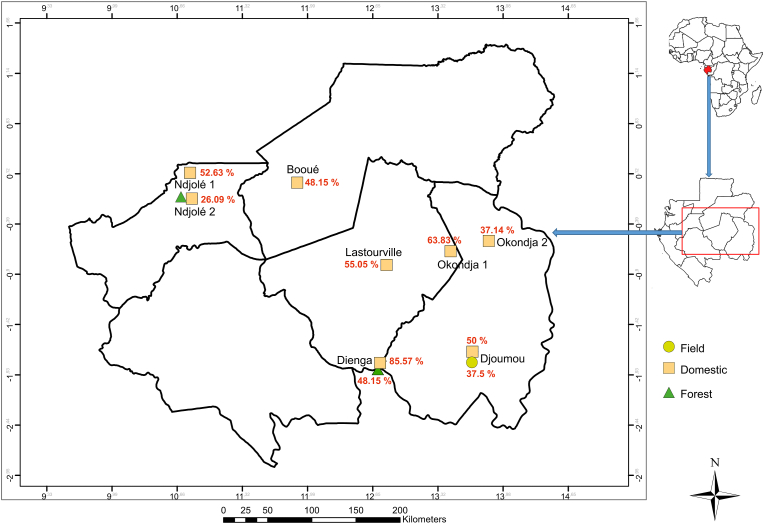
**Location of rodent sampling sites in Gabon.** Squares represent domestic sites, while the circle and triangles indicate field and forest sites, respectively, where rodents were trapped. Red labels correspond to the *Trypanosoma* prevalences detected by qPCR at each site.

The samples analyzed in this study were collected during field sessions conducted between October 2021 and June 2022, following a sampling protocol described in detail in (Mbou-Boutambe et al., 2024; Mbou-Boutambe et al., 2025). Briefly, small mammals were trapped in eight rural sites across four regions of Gabon (Fig. 1). In all sites, the traps were set in houses for three consecutive nights. For three localities (Ndjolé, Dienga and Djoumou) additional sampling was also performed in the forest or in the crop fields close to the villages. A total of 6158 trap-nights yielded 743 rodents, including 667 *Rattus* (89.8%), 10 *Mus musculus domesticus* (1.3%) and 66 individuals of 12 native rodent species (8.9%). Six shrews (*Crocidura* spp.) were also captured but were excluded from analyses. Rodent species were identified by morphometric analyses following (Happold, 2013) and using genetic tools to avoid any ambiguity in the identification (barcoding using partial *cytb* Sanger sequencing) (Nicolas et al., 2012). Sufficient blood (>200 μL) was collected from 528 individuals for molecular screening of *Trypanosoma* spp.

### 2.3 DNA extraction, amplification, sequencing and phylogeny

Blood samples were analyzed using molecular approaches to detect *Trypanosoma* spp. infections. DNA was extracted from approximately 200 μL of whole blood using the DNeasy Blood & Tissue Kit (Qiagen, Courtabœuf, France) following the manufacturer's instructions.

Detection of *Trypanosoma* spp. relied on qPCR amplification of a 131 bp fragment of the 18S rDNA gene on a LightCycler® LC480 instrument (Roche Diagnostics, Meylan, France) following Dobigny et al. (2011) with the following modifications: a final reaction volume of 7 μL including 2 μL of template DNA and no addition of uracil-DNA-glycosylase.

Samples with a Ct value < 40 were considered positive. This threshold was empirically justified because the most late-amplifying sample successfully sequenced (a *Praomys jacksoni* individual) exhibited a Ct value of 39.8, in line with the methodological approach used by (Tatard et al., 2017).

To optimize budgetary and logistical resources, longer SSU (18S) rDNA sequencing was performed using a single 95-well plate. Samples were assigned to the plate based on a strict eco-epidemiological prioritization framework rather than random selection. First, all native rodent individuals that tested positive during the initial short-fragment screening were systematically included. These native host samples were prioritized *a priori* due to their characteristically low parasite loads, as indicated by late qPCR cycle threshold (Ct) values (approx 40). This comprehensive inclusion aimed to maximize the recovery of native host parasite diversity despite inherent technical difficulties.

The remaining wells of the 95-well plate were then filled using a stratified selection of positive *Rattus* samples. To ensure robust spatial and ecological representativeness for this dominant species, these samples were selected across diverse geographical sampling localities rather than at random. Due to biological and technical limitations linked to low parasite DNA yields, some prioritized native host samples either failed to amplify during the subsequent nested PCR steps or yielded low-quality chromatograms that did not meet our stringent quality control thresholds. Consequently, successful sequences could not be recovered for all qPCR-positive native hosts. A comprehensive, transparent breakdown of the total analyzed rodents, qPCR-positive samples, and successfully sequenced individuals by both host species and locality is provided in Tables S1 and 2.

To characterize trypanosome diversity, we first performed a conventional PCR targeting a 400-bp fragment of the 18S rDNA gene following Dobigny et al. (2011) on qPCR-positive samples selected to represent the diversity of rodent species and sampling localities, following Dobigny et al. (2011). Amplification was carried out on a Mastercycler ep-Gradient S thermocycler (Eppendorf) in 25 μL reactions containing 1× buffer (2 mM MgCl_2_), 0.1 mM of each dNTP, 0.5 μM of each primer (TRYPASEQ1 and TRYPASEQ2), 1 U DreamTaq polymerase (ThermoFisher) and 2 μL of template DNA. The thermal profile consisted of 95 °C for 5 min; 40 cycles of 95 °C for 1 min, 55 °C for 45 s and 72 °C for 1 min; and a final extension at 72 °C for 10 min. Five microliters of each product were electrophoresed on 1.5% agarose gels in Tris-acetate-EDTA buffer and sequenced by Eurofins MWG (France).

Second, a trypanosomatid-specific nested PCR targeting a longer of 2100 bp fragment of the 18S rDNA gene, was also performed following (Seward et al., 2017) on some samples (from native and invasive rodents) previously sequenced for the 400 bp fragment. Amplification was performed on 4 μl of DNA. Five mL of each amplicon were run on 1.5% agarose gels in Tris-acetate-EDTA buffer and sequenced by Eurofins MWG (France).

For phylogenetic analysis, the nucleic acid sequences obtained in this study (GenBank accession numbers PZ112831 to PZ112887 for the 400-bp sequences and PZ093096 to PZ093116 for the 2100-bp sequences) were compared with reference sequences from GenBank. These reference taxa, representing various previously characterized studies and geographical regions, were identified using BLAST searches (see Supplementary data Tables S3 and 4).

The two multiple alignments of all sequences (obtained and reference ones) were subsequently performed using the ClustalW tool, as implemented in the MEGA11 software (Tamura et al., 2021).Phylogenetic relationships were inferred using each alignment using a Bayesian inference approach implemented in *MrBayes* v.3.2.2, following optimization of the nucleotide substitution model with *ModelTest* v.3.06. For the dataset concerning the 400 bp sequences, the K2+ G model was identified as the best-fitting model of sequence evolution, whereas the GTR + I + G model was selected for the dataset concerning the 2100 bp sequences. Bayesian analyses were run for five million generations under a covarion model, with sampling every 500 generations. All other parameters were kept at their default values.

We then focused on *R. rattus*, which was largely dominant in our samples (see results) for the following analyses. Statistical analyses were performed using R software (v.3.2.1), *p*-values being considered as significant below a 0.05 level. We then evaluated whether age class and sex of rodents may be related to prevalence levels of *Trypanosoma* in domestic localities. The relationship of *Trypanosoma* infection (presence/absence) with age class and sex was assessed using a generalized linear mixed model (GLMM) assuming a binomial distribution, with locality as a random factor.

## 3 Results

### 3.1 *Trypanosoma* spp. infections

The prevalence of *Trypanosoma* spp. among rodents was assessed based on all qPCR-positive individuals. In total, 299 rodents were positive for trypanosomes. *Trypanosoma*-positive rodents were detected in all studied localities (Table 1). The majority of infected individuals (89%; 267 out of 299) were *R*. *rattus*, with prevalence rates in this species ranging from 21.6% to 85.7% across sites. *Mus musculus* individuals (only captured in Ndjolé 1 and 2) showed an infection prevalence of 42.8% (3 out of 7) in Ndjolé 2, and the only individual captured in Ndjolé 1 was also infected. Native rodents were also found infected with *Trypanosoma* spp. (57.1%; 28 out of 49) at six sites (Dienga-domestic, Dienga-forest, Djoumou-field, Lastourville-domestic, Ndjolé 2-domestic, and Ndjolé-forest: see Table 1). Prevalence ranged from 0% (0/2) in *Lophuromys nudicaudatus* and *Malacomys longicaudatus*, to 100% (6/6) in *Praomys jacksoni*, but sample sizes were generally low in these rodent species. GLMM analyses indicated that adult *R. rattus* individuals were significantly more infected by *T. lewisi* than juvenile ones (*p* = 0.017). No statistical difference was found between females and males (*p* = 0.43).

**Table 1:** **Rodent species sampled in eleven sites of Gabon and number of individuals infected by *Trypanosoma* spp. per species and site, determined by qPCR amplification of a fragment of the 18S rDNA gene.** For each site, an estimate of the trapping effort is provided. For each taxon: number of infected individuals/number of individuals trapped. Exotic species: R. ra: *Rattus* and *M. mus*: *Mus musculus domesticus*. Native species: H.un: *Hybomys univittatus*, H.ae: *Hylomyscus aeta*, L.st: *Lemniscomys striatus*, L.ro: *Lophuromys roseveari*, M. lo: *Malacomys longipes*, M.mi: *Mus minutoides*, O.hy: *Oenomys hypoxanthus*, P.ja: *Praomys jacksoni*, P.mi: *Praomys misonei* and P.pe: *Praomys petteri*.

Site	Booué_domestic	Dienga_domestic	Dienga_forest	Djoumou_field	Djoumou_domestic	Lastourville_domestic	Ndjolé 1_domestic	Ndjolé 2_domestic	Ndjolé_forest	Okondja 1_domestic	Okondja 2_domestic	Total
**Trap. nights**	1314	970	176	463	291	886	330	836	178	588	126	6158
**Total**	99	121	31	9	14	122	50	113	2	130	52	743
**Captures**												
H.un	-	2/2	3/5	-	-	-	-		-	-	-	5/7
H.ae	-	1/1	-	-	-	-	-	-	-	-	-	1/1
L.st	-	0/1	2/8	0/2	-	1/1	-	-	-	-	-	3/12
L.nu	-	-	-	0/2	-	-	-	-	-	-	-	0/2
L.ro	-	2/2	6/9	-	-	-	-	-	-	-	-	8/11
M.lo	-	-	0/2	-	-	-	-	-	-	-	-	0/2
M.mi	-	1/1	-	-	-	-	-	1/1	-	-	-	2/2
M.mu	-	-	-	-	-	-	1/1	3/7	-	-	-	4/8
O.hy	-	-	1/2	-	-	-	-	-	-	**-**	-	1/2
P.ja	-	5/5	-	-	-	1/1	-	-	-	-	-	6/6
P.mi	0/1	-	-	1/1	-	-	-	-	-	-	-	1/2
P.pe	-	0/1	1/1	-	-	-	-	-	-	-	-	1/2
R.ra	39/80	72/84	-	1/2	7/14	58/107	9/18	8/37	-	60/94	13/35	**267/471**
	(48.75 ± 8.76)	(85.71 ± 6.29)				(54.21 ± 10.10)	(50 ± 4.84)	(21.62 ± 4.91)		(63.83 ± 9.13)	(100 ± 37.14)	**(56.69 **±** 5.60)**
**Total**	39/81	**83/97**	13/27	2/7	7/14	60/109	10/19	12/45	-	60/94	13/35	**299/528**
	(48.15 ± 8.81)	**(85.57 **±** 6.78)**	(48.15 ± 5.09)			(55.05 ± 10.18)	(100 ± 4.27)	(26.67 ± 5.81)		(63.83 ± 9.13)	(100 ± 37.14)	**(56.63 **±** 22.32)**

### 3.2 Phylogenetic positioning of *Trypanosoma* sequences from Gabon

**Fig. 2:**
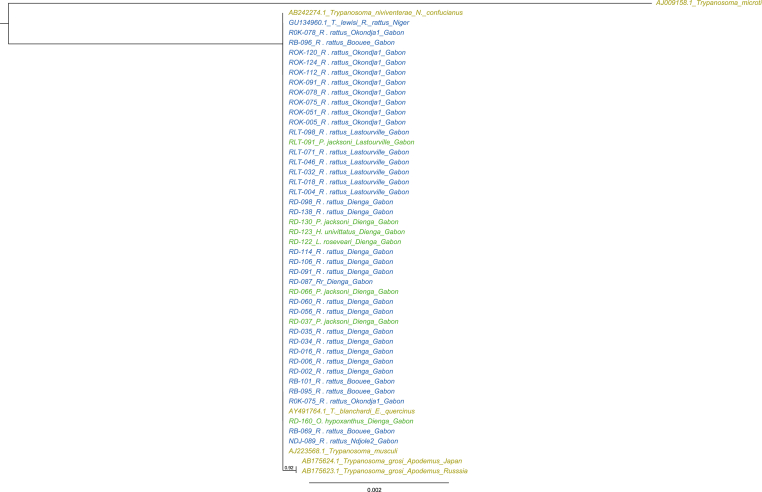
Bayesian phylogenetic tree based on 47 partial 18S rRNA gene sequences (361 bp) of *Trypanosoma* spp. Label of sequences from this study are colored in blue (*R. rattus*) and green (native rodents); other sequences of the subgenus *Herpetosoma* retrieved from Genbank are indicated in brown. Bootstrap values > 0.70 are shown at nodes. Country abbreviations are listed in Supplementary Table 3. The sequence of *T. microti* (AJ009158) was used as the outgroup.

We sequenced 400 bp of the SSU rDNA gene for 95 samples detected as qPCR-positive for trypanosomes (21 from native rodents; 73 from *R. rattus* and one from *Mus musculus domesticus*) in our study. From this dataset, 70 sequences (63 from *Rattus* and 7 from other local rodent species) were recovered (Fig. 2, Supplementary data Table 1). The obtained phylogenetic tree based on an alignment of 361 bp shows that all sequences from Gabon belong to one clade (Fig. 2). The tree does not enable to differentiate our sequences from reference sequences (retrieved from GenBank), of species belonging to the *T. lewisi* subclade (Votýpka et al., 2022) such as *T. blanchardi, T. musculi*, *T. niviventerae*, and *T. rabinowitschae*, and of others species (like *T. grosi*, *T. kuseli*, *T. microti*, and *T. otospermophili*) of the *Herpetosoma* subgenus (Fig. 2).

**Fig. 3:**
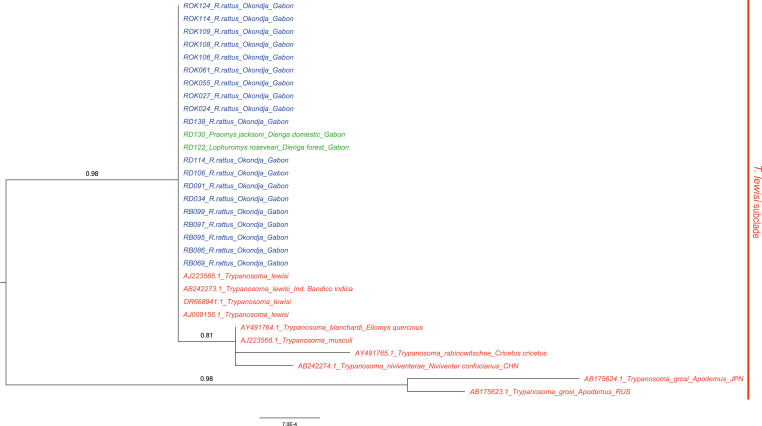
Bayesian phylogenetic tree based on 31 partial 18S rRNA sequences (1981 bp) of *Trypanosoma lewisi* subclade. Label of sequences from this study are colored in blue (*Rattus*) and green (native rodents). In red: sequences from the GenBank database associated with the *Trypanosoma lewisi* subclade. Bootstrap values > 0.7 are shown at nodes. Country abbreviations are listed in Supplementary Table 4. The sequences of *T. grosi* were used as the outgroup.

Twenty-one samples (19 from *R. rattus*, 2 from native *P. jacksoni* and *L*. *roseveari*) were sequenced for a longer fragment of the 18S rDNA gene (See Supplementary data Table 2). All sequences from Gabon clustered tightly within the *T. lewisi* subclade, corresponding to genotype AF05b with 100% nucleotide identity, which is the genotype recognized as representing *T. lewisi* sensu stricto (Fig. 3) (Babyesiza et al., 2024).

## 4 Discussion

Rodent communities in Gabonese villages were largely dominated by the exotic *R. rattus*, a species widely recognized as the main reservoir and dispersal host of the zoonotic trypanosome *T. lewisi* across Sub-Saharan Africa. In this study, we investigated the prevalence and diversity of *Trypanosoma* spp. infecting rodents across eight rural localities in Gabon, encompassing both domestic and non-domestic environments. Contrary to expectations, *Trypanosoma* prevalence did not differ significantly between rodents sampled in villages (57%; 284/494) and those captured in crops or forests (44%; 15/34). This absence of a significant difference should be interpreted cautiously, given the strong imbalance in sample sizes between habitats. Nevertheless, this pattern may reflect the high permeability of habitat boundaries in rural African landscapes. In rural Gabon, households are often embedded within a mosaic of croplands, fallows, secondary forests, allowing rodents to move freely between domestic, peri-domestic, and non-domestic environments (Mangombi-Pambou et *al*., 2023). Such connectivity likely facilitates interactions and parasite transmission among rodent species.

### 4.1 High prevalence of *Trypanosoma* spp. in Gabonese rodents

Overall, more than half of the rodents screened (56.6%) were positive for *Trypanosoma* spp., with prevalence varying markedly among sites (26.1-85.6%). These values are among the highest reported to date in African rodent communities. By comparison, prevalence rates reported in previous studies ranged from 14 to 50% in villages in Senegal (Cassan et al., 2018), and approximately 15% in villages and cities in Niger and Nigeria (Tatard et al., 2017). Lower prevalences were also reported in East Africa, with rates of 22.9% in Kenya, 18.3% in Ethiopia, 5.8% in Tanzania (Votýpka et *al*., 2022), and up to 23% in Uganda (Babyesiza et al., 2024). The particularly high prevalence observed in rural Gabon may reflect intense local transmission linked to high rodent densities, close contact between hosts and vectors, and/or the overwhelming dominance of *R. rattus* in domestic environments.

### 4.2 Central role of *Rattus* in parasite transmission

The vast majority of infected rodents in our study were *R. rattus* (89%). All sequences obtained from *R. rattus* clustered within *T*. *lewisi* sensu stricto (genotype AF05b), in agreement with previous studies across Africa (Cassan et al., 2018; Dobigny et al., 2019; Tatard et al., 2017). These results reinforce the view that *R. rattus* acts as the primary reservoir host of *T. lewisi* in anthropized environments.

Although human infections by *T. lewisi* remain rare, several atypical cases of human trypanosomiasis caused by this species have been reported worldwide (Howie et al., 2006; Verma et al., 2011). Given the high abundance of *R. rattus* within households in rural Gabon, the intense circulation of *T. lewisi* documented here may represent a non-negligible risk of human exposure.

### 4.3 Limited parasite diversity and presence of *T. lewisi* native rodent species

Unlike the broader diversity of trypanosome species reported by Votýpka et *al*. (2022), all sequences recovered in this study corresponded to *T*. *lewisi* sensu stricto *(*genotype AF05b*)*. This apparent lack of diversity is likely driven by the strong dominance of *Rattus* in our sampling and the relatively low number of native rodent species captured. In contrast, Votýpka et *al*. (2022) surveyed a wide range of sylvatic rodents across multiple East African countries and habitats, increasing the likelihood of detecting trypanosomes with diverse host ranges and transmission cycles. Furthermore, the environmental scope of our study was restricted to rural and peri-domestic settings, which are typically characterized by flea-mediated transmission cycles dominated by *T. lewisi*. More heterogeneous and less anthropized landscapes, such as those sampled in East Africa, are likely to support additional vectors and transmission routes, contributing to higher parasite diversity. The low success rate in amplifying the long 2100 bp fragment (21/299) further highlighted the technical challenge of handling field samples, likely due to low parasitemia in some hosts, especially native rodents. For future wild rodent surveys, using intermediate-length 18S rDNA fragments could offer a powerful trade-off between amplification feasibility and phylogenetic resolution within the *Herpetosoma* subgenus.

Despite the dominance of *R. rattus*, *T. lewisi* was also detected in two native rodent species (*Praomys jacksoni* and *Lophuromys roseveari*), with sequences identical to those obtained from black rats. This finding aligns with numerous reports of *T. lewisi* infecting a wide range of native African rodents, including species of *Acomys*, *Mastomys*, *Arvicanthis*, *Cricetomys*, *Lophuromys*, *Praomys*, and *Graphiurus* across West, Central, and East Africa (Dobigny et al., 2011; Ortiz et al., 2018; Salzer et al., 2016; Tatard et al., 2017; Votýpka et *al*., 2022). While these observations are compatible with the hypothesis of potential spill-over events from *R. rattus* to native hosts, our limited sample size for native rodents precludes any robust conclusion regarding the direction or frequency of these transmissions. Further studies with balanced sampling are required to determine whether *T. lewisi* is maintained through sustained transmission within native populations or results from opportunistic spill-overs. Several authors have proposed that *R. rattus* functions as the main reservoir, while native species act as opportunistic or incidental hosts (Hoare, 1972, Sato et al., 2007). The ecological consequences of such spill-over events on native species should not be underestimated. The well-documented case of Christmas Island illustrates how the introduction of *T. lewisi*, by invasive *R. rattus* likely contributed to the extinction of two endemic rat species (*Rattus macleari* and *Rattus nativitatis*) (Wyatt et al., 2008). Although such extreme outcomes may be rare, they highlight the vulnerability of naïve hosts and the potential for invasive species to disrupt native parasite-host dynamics.

## 5 Conclusion

Our study provides the first molecular characterization of *Trypanosoma lewisi* (AF05b) circulating in *R. rattus* from rural Gabon, revealing a widespread distribution and remarkably high prevalence levels in the invasive *Rattus*. Overall, our results demonstrate that the transmission dynamics of *T. lewisi* in Gabonese villages are largely driven by the synanthropic ecology and overwhelming dominance of *R. rattus*, which acts as the primary reservoir host in these anthropized systems. Although *T. lewisi* DNA was also detected in some native rodent species, our sampling imbalance limits further conclusions regarding the frequency or direction of potential spill-over events. These findings emphasize the importance of monitoring invasive rodent populations as major parasite amplifiers, and underscore the need for future targeted studies to clarify the epidemiological role and trypanosome diversity of native African rodents at the wildlife-invasive interface.

## Data availability statement

The original contributions presented in this study are included in the article; for further inquiries, please contact the corresponding author. The partial genomic sequences/coding genes of the studied trypanosomes are available in the National Center for Biotechnology Information (NCBI) GenBank database under the accession numbers listed in this article. Access to FASTA files is available upon request.

## Funding sources

This study was funded by ANR MICETRAL (Project number ANR-19-CE35-0010).

## CRediT authorship contribution statement

**Clark Mbou-Boutambe:** Data curation, Formal analysis, Investigation, Methodology, Validation, Visualization, Writing – original draft. **Larson Boundenga:** Investigation, Project administration, Resources, Supervision, Validation, Visualization. **Carine Brouat:** Conceptualization, Project administration, Resources, Supervision, Validation, Visualization, Writing – review & editing. **Philippe Gauthier:** Methodology, Writing – review & editing. **Odile Fossati-Gaschignard:** Formal analysis. **Fanny Degrugillier:** Formal analysis. **Thierry Audrey Tsoumbou:** Investigation. **Barthélemy Ngoubangoye:** Investigation. **Virginie Rougeron:** Project administration, Resources, Supervision, Validation, Writing – review & editing. **Laurent Granjon:** Project administration, Resources, Supervision, Validation, Writing – review & editing. **Franck Prugnolle:** Conceptualization, Data curation, Funding acquisition, Project administration, Resources, Supervision, Validation, Visualization, Writing – review & editing.

## Conflict interest

The authors declare no conflict of interest.
