# Supplementary material for: Trypanosoma lewisi infections in Rattus rattus from rural ecosystems in Gabon
Source: Int J Parasitol Parasites Wildl. 2026 Jun 20;30:101257. doi: 10.1016/j.ijppaw.2026.101257 (PMC13331795; doi:10.1016/j.ijppaw.2026.101257)
Supplement: Multimedia component 1 [file mmc1.docx]

# Supplementary data.

**Supplementary data Table 1.** Sequencing results of the 400-bp fragment of the 18S rDNA gene for the 95 analyzed individuals.

| **Small mammals** | **Min (Ct)** | **DNA sequence *Trypanosoma* spp.** | **Mean (Ct)** |
| --- | --- | --- | --- |
| *Hybomys univittatus* | 33-37 | 1 | 35.2 |
| *Hylomyscus aeta* | 33-35 | 0 | 34.3 |
| *Lemniscomys striatus* | 32-37 | 0 | 36.2 |
| *Lophuromys roseveari* | 29-40 | 1 | 35.4 |
| *Lophuromys nudicaudatus* | - | - | **-** |
| *Malacomys longipes* | - | - | **-** |
| *Mus musculus domesticus* | 31-42 | 0 | 37.5 |
| *Mus minutoides* | 36-38 | 0 | 36.8 |
| *Oenomys hypoxanthus* | 28-30 | 1 | 29.2 |
| *Praomys jacksoni* | 31-40 | 4 | 34.5 |
| *Praomys misonnei* | 34-36 | 0 | 35.3 |
| *Praomys petteri* | 37-38 | 0 | 36.9 |
| *Rattus rattus* | 14-49 | 63 | 32.5 |
| **TOTAL** |  | 70 |  |

**Supplementary data Table 2.** Sequencing results of the 2100-bp fragment of the 18S rDNA gene.

| **Rodents** | **Number**  **Screened** | **Number successfully sequenced** | **Failed**  **samples** | **Min**  **(Ct)** | **Mean**  **(Ct)** |
| --- | --- | --- | --- | --- | --- |
| *Hybomys univittatus* | 1 | 0 | 1 | 33-37 | 33.855 |
| *Lophuromys roseveari* | 1 | 1 | 0 | 29-40 | 34.225 |
| *Oenomys hypoxanthus* | 1 | 0 | 1 | 28-30 | 29.22 |
| *Praomys jacksoni* | 4 | 1 | 3 | 31.725-39.8 | 35.192 |
| *Rattus rattus* | 46 | 19 | 27 | 14.95-32.635 | 20.923 |
| **Total** | 53 | 21 | 32 |  |  |

**Supplementary data Table 3**: *Trypanosoma* sequences used as reference to construct the phylogenetic tree (see Fig. 2). For each sequence, the accession number, the host and the country where the parasite was collected is provided.

| Access number | species | Host | Country |
| --- | --- | --- | --- |
| OR452743 | *Trypanosoma sp.* | *Mus bufo* | Uganda |
| AY491765 | *T. rabinowitschae* | *Cricetus cricetus* | NA |
| MZ703253 | *Trypanosoma sp.* | *Lygosoma brevicaudus* | Ethiopia |
| MZ703258 | *Trypanosoma sp.* | *Myomyscus brockmani* | Ethiopia |
| MZ703256 | *Trypanosoma* sp*.* | *Acomys Percivali* | Ethiopia |
| MK603814 | *T. noyesi* | *Sylvisorex anodontis* | Thailand |
| OP010076 | *T. noyesi* | *Indosorex asperulus* | Thailand |
| OP861673 | *Trypanosoma* sp. | *Sylvisorex anodontis* | Thailand |
| MZ703227 | *Trypanosoma* sp. | *Acomys mullah* | Ethiopia |
| OR452747 | *Trypanosoma* sp. | *Acomys Percivali* | Uganda |
| KF054111 | *Trypanosoma* sp. | *Indosorex asperulus* | Thailand |
| MZ703239 | *Trypanosoma* sp. | *Acomys muzei* | Zambia |
| AB242275 | *Trypanosoma* sp. | *Microtus montebelli* | Japan |
| AJ009158 | *T. microti* | *NA* | NA |
| AB175626 | *T. kuseli* | *Pteromys volans* | NA |
| AB190228 | *T. otospermophili* | *Spermophilus columbianus* | Colombia |
| AB242274 | *T. niviventerae* | *Niviventer confucianus* | NA |
| AJ223568 | *T. musculi* | *NA* | NA |
| AY491764 | *T. blanchardi* | *Eliomys quercinus* | NA |
| GU134960 | *T. lewisi* | *Rattus rattus* | Niger |
| MZ703241 | *Trypanosoma* sp. | *Gerbillus vicinus* | Tanzania |
| MZ703245 | *Trypanosoma* sp. | *Gerbillus vicinus* | Tanzania |
| OR452748 | *Trypanosoma sp.* | *Crocidura hildegardeae* | Uganda |
| MZ703246 | *Trypanosoma sp.* | *Leontopithecus rosalia* | Tanzania |
| MZ703235 | *Trypanosoma sp.* | *Acomys* | Ethiopia |
| MZ703236 | *Trypanosoma sp.* | *Lophuromys* | Ethiopia |
| OR452750 | *Trypanosoma sp.* | *Lophuromys* | Uganda |
| OR452745 | *Trypanosoma sp.* | *Mus bufo* | Uganda |
| AB175623 | *T. grosi* | *Apodemus* | Russia |
| AB175624 | *T. grosi* | *Apodemus* | Japan |
| LC830952 | *T. congolense* | *NA* | NA |
| AF306776 | *T. brucei* | *NA* | NA |
| AF306777 | *T. brucei* | *NA* | NA |
| LC521915 | *T. evansi* | *NA* | NA |
| LC521916 | *T. evansi* | *NA* | NA |
| JN673387 | *T. simiae* | *NA* | *NA* |
| JF746736 | *T. cruzi* | *NA* | *NA* |

**Supplementary data Table 4**: *Trypanosoma* sequences used as reference to construct the phylogenetic tree (see Fig. 3). For each sequence, the accession number, the host and the country where the parasite was collected is provided.

| Access number | species | Host | Country |
| --- | --- | --- | --- |
| AB175623 | *T. grosi* | *Apodemus* | Russia (RUS) |
| FJ694763 | *T. grosi* | *Apodemus* | China (CHN) |
| AB175624 | *T. grosi* | *Apodemus* | Japan (JPn) |
| OR668941 | *AF05b* | *Mus triton* | Uganda (UGA) |
| MZ703216 | *AF05b* | *Mus triton* | Kenya (KEN) |
| AJ009156 | *T. lewisi* | *Rattus norvegicus* | United Kingdom |
| AB242273 | *T. lewisi* | *Bandico indica* | India (IND) |
| GU252209 | *T. lewisi* | *Alouatta fusca* | Brazil |
| AY491765 | *T. rabinowitshae* | *Cricetus cricetus* | *NA* |
| AY491764 | *T. blanchardi* | *Eliomys quercinus* | *NA* |
| AJ223568 | *T. musculus* | *NA* | NA |
| AB242274 | *T. niviventerae* | *Niviventer conficianus* | China (CHN) |
